# Combining BET and HDAC inhibitors synergistically induces apoptosis of melanoma and suppresses AKT and YAP signaling

**DOI:** 10.18632/oncotarget.4242

**Published:** 2015-06-05

**Authors:** Anja Heinemann, Carleen Cullinane, Ricardo De Paoli-Iseppi, James S. Wilmott, Dilini Gunatilake, Jason Madore, Dario Strbenac, Jean Y. Yang, Kavitha Gowrishankar, Jessamy C. Tiffen, Rab K. Prinjha, Nicholas Smithers, Grant A. McArthur, Peter Hersey, Stuart J. Gallagher

**Affiliations:** ^1^ Melanoma Research Group, Kolling Institute of Medical Research, University of Sydney, St Leonards NSW, Australia; ^2^ Melanoma Institute of Australia, North Sydney, NSW, Australia; ^3^ Melanoma Immunology and Oncology Group, Centenary Institute, University of Sydney, Camperdown, NSW, Australia; ^4^ Translational Research Laboratory, Peter MacCallum Cancer Centre, Melbourne, VIC, Australia; ^5^ Sydney Medical School, University of Sydney, Sydney, NSW, Australia; ^6^ School of Mathematics and Statistics, University of Sydney, Sydney NSW, Australia; ^7^ Epinova Discovery Performance Unit, GlaxoSmithKline, Stevenage, United Kingdom

**Keywords:** panobinostat, I-BET151, melanoma, bromodomain, epigenetic

## Abstract

Histone acetylation marks have an important role in controlling gene expression and are removed by histone deacetylases (HDACs). These marks are read by bromodomain and extra-terminal (BET) proteins and novel inhibitiors of these proteins are currently in clinical development. Inhibitors of HDAC and BET proteins have individually been shown to cause apoptosis and reduce growth of melanoma cells. Here we show that combining the HDAC inhibitor LBH589 and BET inhibitor I-BET151 synergistically induce apoptosis of melanoma cells but not of melanocytes. Induction of apoptosis proceeded through the mitochondrial pathway, was caspase dependent and involved upregulation of the BH3 pro-apoptotic protein BIM. Analysis of signal pathways in melanoma cell lines resistant to BRAF inhibitors revealed that treatment with the combination strongly downregulated anti-apoptotic proteins and proteins in the AKT and Hippo/YAP signaling pathways. Xenograft studies showed that the combination of inhibitors was more effective than single drug treatment and confirmed upregulation of BIM and downregulation of XIAP as seen *in vitro*. These results support the combination of these two classes of epigenetic regulators in treatment of melanoma including those resistant to BRAF inhibitors.

## INTRODUCTION

Dysregulation of chromatin structure is a frequent event in melanoma [1–3] and underlies many aspects of melanoma biology including resistance to targeted therapies [4, 5] and melanoma invasiveness [6]. The regulation of chromatin structures is largely under the control of several protein classes that modify histones. These include proteins that add acetyl, methyl or other groups to histones (writers) or “erasers” such as histone deacetylases (HDACs) and demethylases which remove these groups. The writers and erasers establish what is referred to as a “histone code” that is “read” by a third class of proteins that recognize the histone code and act to focus large protein complexes including transcription factors to those sites [7, 8]. These protein complexes determine the gene expression status such as repression or activation and may differ depending on the particular tissues involved [9, 10].

We have shown previously that pan-HDAC inhibitors can induce apoptosis in melanoma that is associated with upregulation of BIM, BAX and BIK and downregulation of Bcl-XL and XIAP [5, 11, 12]. Such inhibitors were also strongly synergistic with selective BRAFi in induction of apoptosis of melanoma [4, 5]. Particular interest has focused on the development of inhibitors against a highly conserved class of “reader” proteins referred to as bromodomain and extra-terminal (BET) proteins. Members of the BET family, which consists of BRD2, BRD3, BRD4 and the testis specific BRDT, have two bromodomains in the N-terminal region which bind to acetylated lysines in histones and a C-terminal (CT) region which binds to transcription elongation factors (TEFs) [13, 14]. The bromodomains act to target protein complexes to particular chromosomal regions involved in gene transcription and act as coregulators of transcription [14–16]. We have previously shown that the BET protein inhibitor I-BET151 described by Nicodeme et al. (2010) exerts anti-melanoma activity by inducing apoptosis, cell cycle arrest and inhibition of tumor growth of xenografts *in vivo* [17, 18]. Additionally I-BET151 has strong inhibitory effects on activation of NF-kB [19].

In the present study we have examined whether combining the HDAC inhibitor LBH589 (panobinostat) and the BET protein inhibitor I-BET151 can potentiate the changes seen when the inhibitors are used as single agents. We report that combination of these two inhibitors has strong synergistic effects in induction of apoptosis, cell cycle arrest and against growth of melanoma xenografts. Moreover apoptosis was mediated by the mitochondrial, caspase-dependent pathway and involved downregulation of the AKT and Hippo/YAP signaling pathway.

## RESULTS

### Combined treatment with I-BET151 and LBH589 synergistically induces apoptosis in melanoma cells

**Figure 1:**
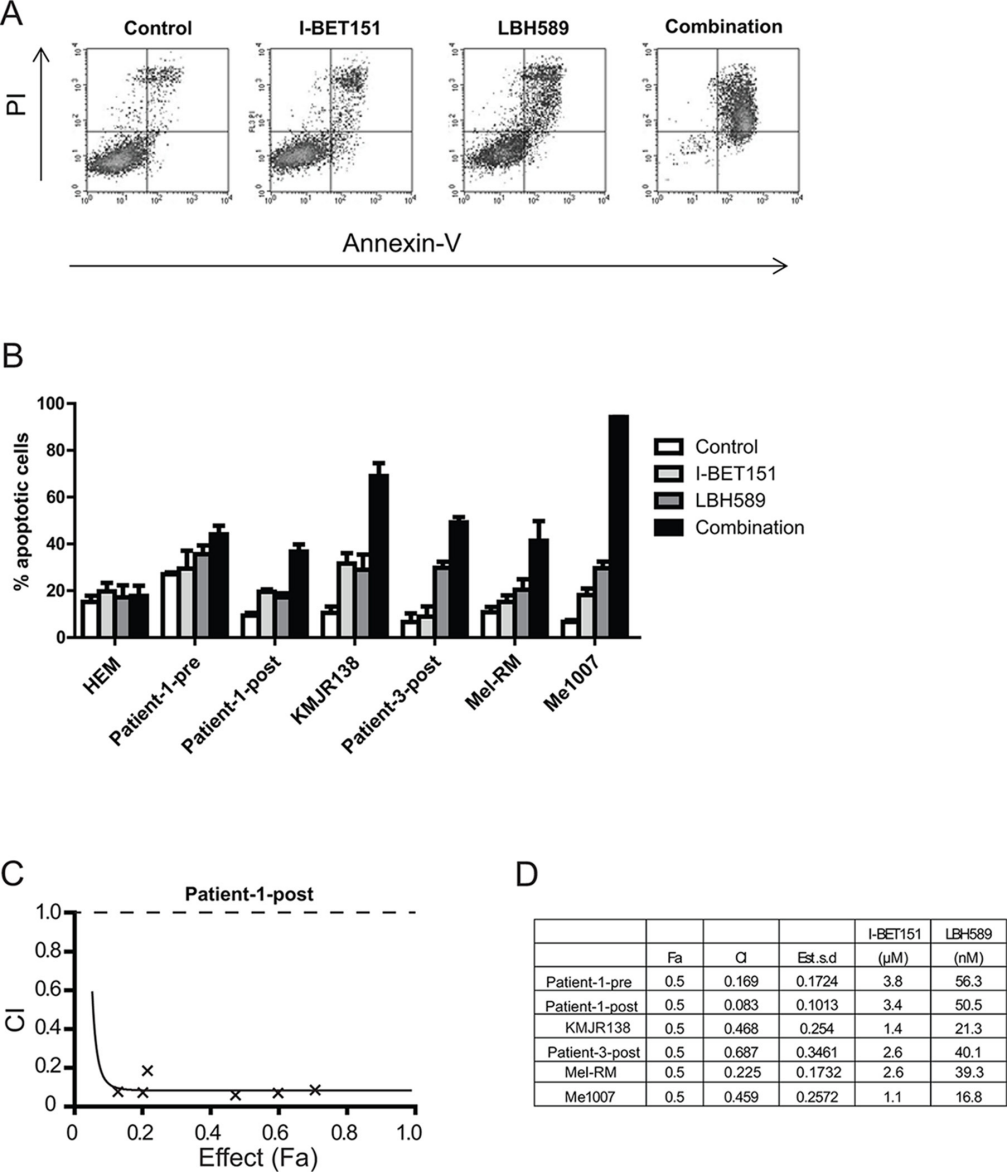
Combination of I-BET151 and LBH589 synergistically induces apoptosis in melanoma cells **A.** Me1007 melanoma cells were treated with 2 μM I-BET151, 30 nM LBH589, combination or control for 48 h. Induction of apoptosis was determined by staining with Annexin-V/PI and flow cytometry analysis. **B.** Histogram represents mean (± SEM) of *n* = 3 experiments of different melanoma cell lines and melanocytes (HEM) drug-treated as described above. Combination treatment significantly induced apoptosis (*p* < 0.05) compared to single drug treatment in all tested melanoma cell lines. **C.** Combination index (CI) of the I-BET151 and LBH589 co-treatment are plotted at increasing drug concentration and fractional effect. CI < 1.0 indicates synergistic interaction. A representative Fa-CI plot (Chou-Talalay plot) for Patient-1-post cells is shown. **D.** CI values for different melanoma cell lines at a fractional effect (Fa) of 0.5 (dose required to kill 50% of cells). CI experiments were performed twice.

To determine whether combined treatment of I-BET151 and LBH589 can potentiate sensitivity of melanoma cells to apoptosis we examined the cytotoxic capacity of both inhibitors in a panel of melanoma cell lines. Dose response curves in a number of cell lines revealed dose-dependent cytotoxicity of the drugs individually or in combination (Supplementary Figure 1A). For subsequent experiments, 2 μM I-BET151 and 30 nM LBH589 were chosen as these concentrations were only slightly toxic individually, but highly cytotoxic in combination. Melanoma cells were treated with these concentrations for 48 h before apoptosis was measured by Annexin-V/PI staining. As shown in Figure 1A single drug treatment of Me1007 cells with I-BET151 or LBH589 showed slight induction of Annexin-V/PI positive cells when compared to DMSO treated cells. Treatment with a combination of both inhibitors markedly increased cell death. The same effect could be shown in other tested cell lines including melanoma cell lines from patients resistant to treatment with the BRAFi vemurafenib (Patient-1-post and Patient-3-post) which were relatively resistant to both drugs alone (Figure 1B). To test if the induction of apoptosis was synergistic rather than merely additive, we performed a combination index (CI) study and calculated synergy using CalcuSyn software. A CI less than 1.0 was obtained in all tested cell lines, indicating a synergistic interaction of both inhibitors with Patient-1-post cells showing the strongest synergistic effect (Figure 1C, 1D).

Studies on the melanoma cell growth showed that the combination of I-BET151 and LBH589 inhibited cell growth and resulted in changes in cell morphology characterized by enlarged and flattened cell bodies (Supplementary Figure 2A). Cell cycle analysis showed the expected sub-G1 population associated with apoptosis and an increase in cells with either 2N DNA content or 4N DNA content, suggestive of arrest in G_0–1_ or G_2-M_ respectively (Supplementary Figure 2B–2C). Alone, I-BET151 treatment predominantly increased the percentage of melanoma cells with 2N DNA content (G_0–1_ phase) while reducing the percentage of S-phase cells. LBH589-treated cells increased the proportion of cells with 4N DNA content. This increase in cells with 4N DNA content may indicate cells arrested in G_2-M_ or cells which have failed to undergo cytokinesis and then arrested in G_1_ but with a 4N DNA content. A similar increase in cells with 4N DNA content was observed in combination-treated cells (except Patient-1-post) suggesting that this growth inhibitory effect is mostly a result of LBH589 inhibitor treatment. Treatment with I-BET151 increased the 4N population in melanocytes. Cell cycle arrest was associated with increases in the cell cycle inhibitor p21 (Supplementary Figure 2D) which was shown previously to be responsible for cell cycle arrest by I-BET151 [17]. Taken together, these results indicate that the combination of I-BET151 and LBH589 synergistically induces apoptosis and cell cycle arrest in melanoma, even in cells with acquired resistance to BRAF inhibitors.

### Apoptosis induced by co-treatment with I-BET151 and LBH589 is caspase dependent and associated with mitochondrial depolarization

**Figure 2:**
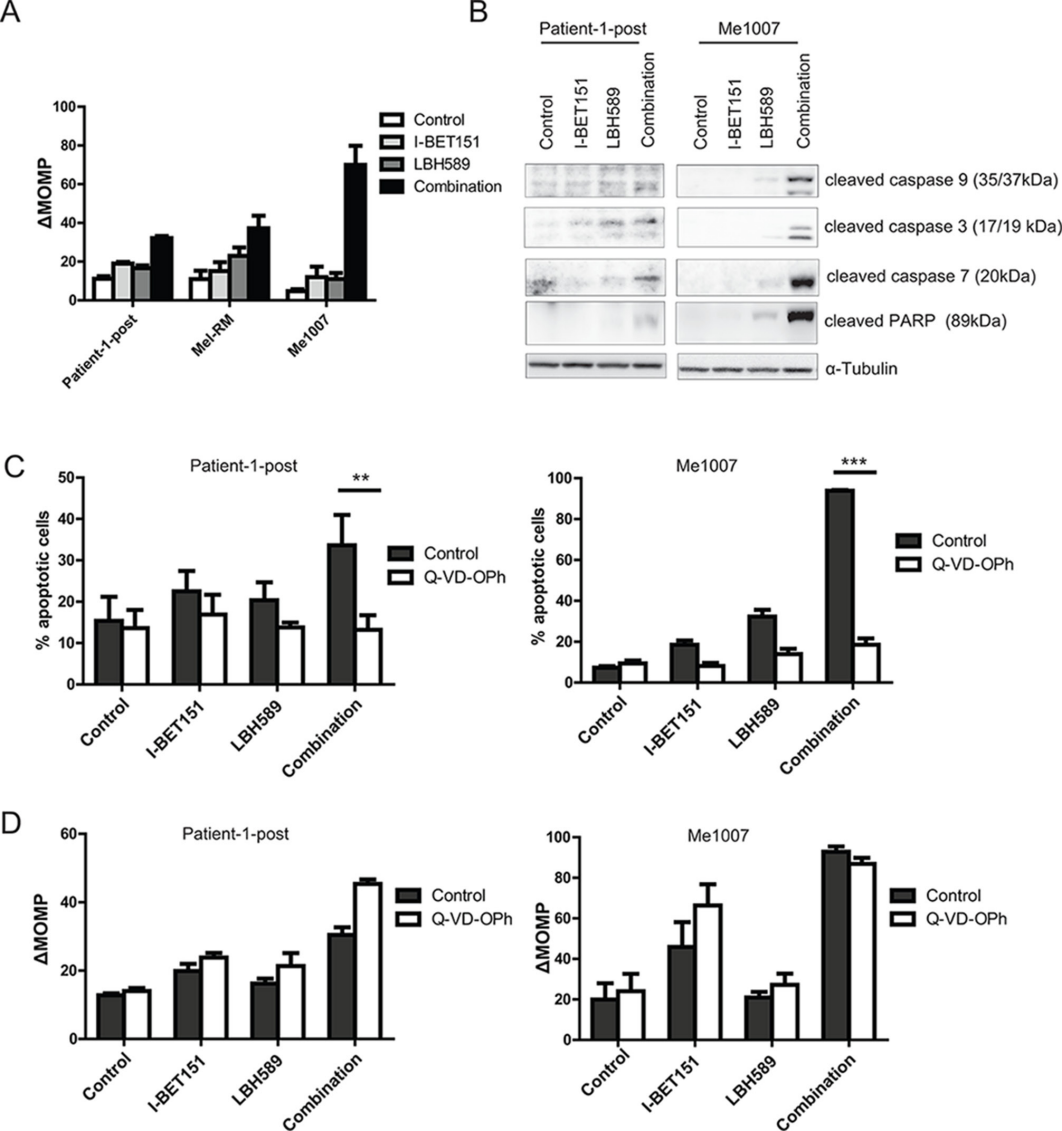
Combination-induced apoptosis is triggered by enhanced mitochondrial depolarization and caspase expression **A.** Loss of mitochondrial outer membrane potential (ΔMOMP) monitored by flow cytometry of JC-1 staining of melanoma cells either treated with 2 μM I-BET151, 30 nM LBH589, combination or DMSO for 48 h is shown (*n* = 3, bars : ± SEM). **B.** Total protein lysates of drug-treated cells for 24 h were analyzed for cleavage of caspases by western blot. α-Tubulin was used as loading control. **C.** Patient-1-post and Me1007 cells were treated with drugs as described before for 48 h. To inhibit caspase activity, melanoma cells were pretreated with 10 μM of caspase inhibitor Q-VD-OPh for 30 minutes before drug treatment. Extent of cell death was assessed using Annexin-V/PI staining. **D.** Mitochondrial depolarization was measured using JC-1. Mean (± SEM) of *n* = 3 experiments is shown.

The presence of Annexin-V positive, PI-negative cells following combined drug treatment (Figure 1A) was suggestive of apoptosis – a form of cell death that may be mediated by mitochondrial depolarization or direct activation of caspases by cytoplasmic membrane bound death receptors. We examined whether the intrinsic mitochondrial pathway was involved by use of the cell permeant dye JC-1 to measure loss of mitochondrial membrane potential (ΔMOMP) in drug-treated melanoma cells. Flow cytometry data revealed that single drug treatment only slightly induced mitochondrial depolarization in melanoma cells (Figure 2A). However treatment with the combination markedly increased mitochondrial depolarization in the Patient-1-post, Mel-RM and Me1007 cells. Using western blotting, we observed a clear increase in cleavage of effector caspases 3, 7 and 9 and caspase substrate PARP following combination drug treatment of Me1007 cells and the vemurafenib-resistant line Patient-1-post (Figure 2B). To investigate whether apoptosis was indeed caspase dependent, cells were pre-treated with the pan-caspase inhibitor Q-VD-OPh. Caspase inhibition completely prevented apoptosis in Patient-1-post and Me1007 cells (Figure 2C). In contrast, Q-VD-OPh treatment did not prevent mitochondrial depolarization in either cell line, indicating that combination of I-BET151 and LBH589 induces an initial caspase-independent loss of mitochondrial depolarization (Figure 2D) followed by caspase dependent apoptosis.

### Induction of apoptosis requires expression of pro-apoptotic BIM

**Figure 3:**
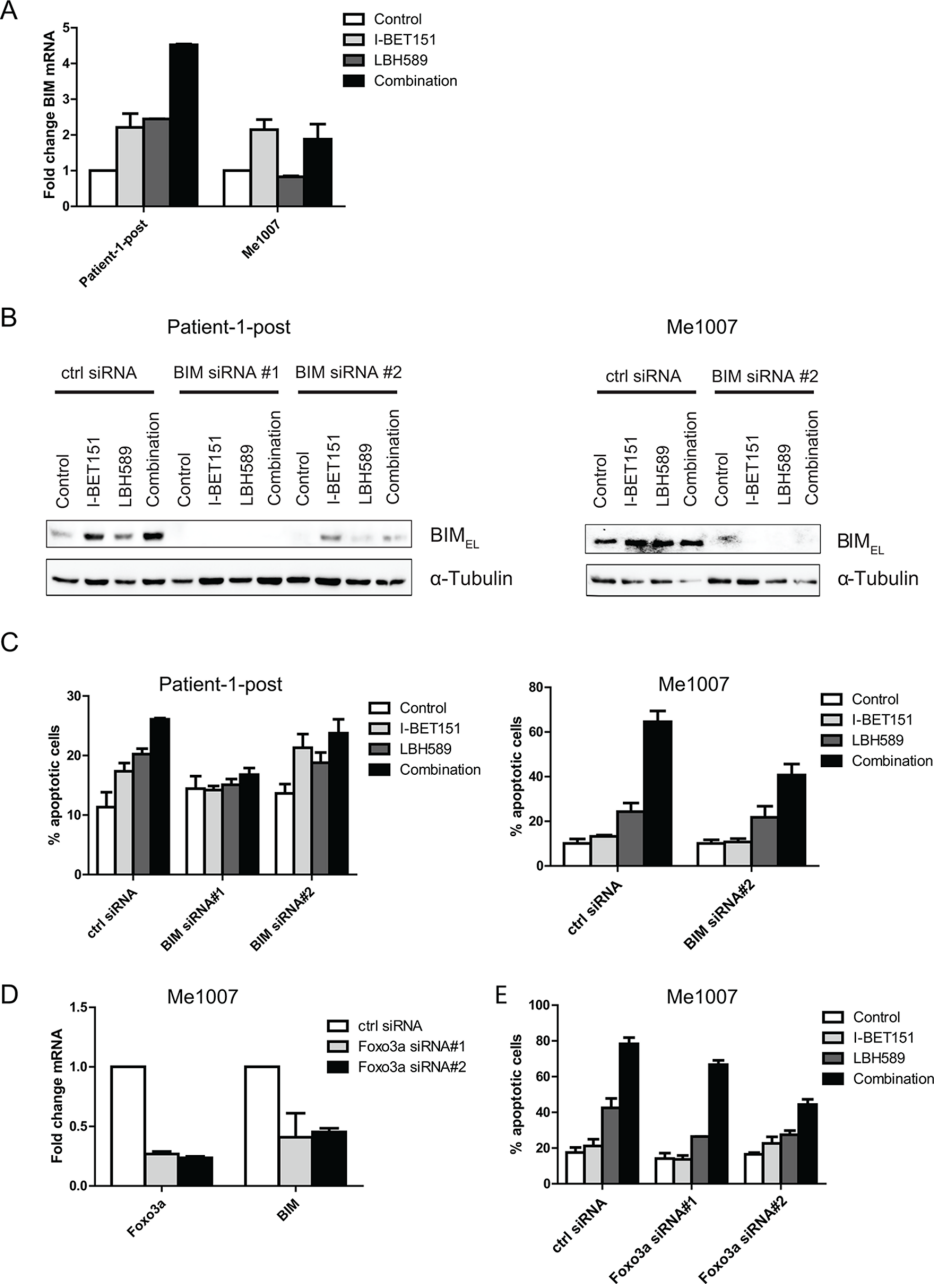
Combination-induced apoptosis requires expression of pro-apoptotic BIM **A.** Induction of BIM mRNA expression level in Patient-1-post and Me1007 cells was determined by qRT-PCR. Mean expression levels (± SEM) of *n* = 2 experiments are presented. **B.** Knockdown of BIM protein expression by siRNA was performed in Patient-1-post and Me1007 cells. After 24 h, transfected cells were treated with drugs as described before and incubated for a further 48 h. Expression of the BIM_EL_ isoform is shown and α-Tubulin served as internal control. **C.** Knockdown of BIM reduces apoptosis in combination-treated in both cell lines. Mean (± SEM) of *n* = 3 experiments is shown. **D.** FOXO3a and BIM mRNA expression level analyzed by qRT-PCR are reduced upon siRNA-mediated knockdown of FOXO3a in Me1007 cells. **E.** Knockdown of FOXO3a reduces percentage of apoptosis in combination-treated Me1007 cells.

**Figure 4:**
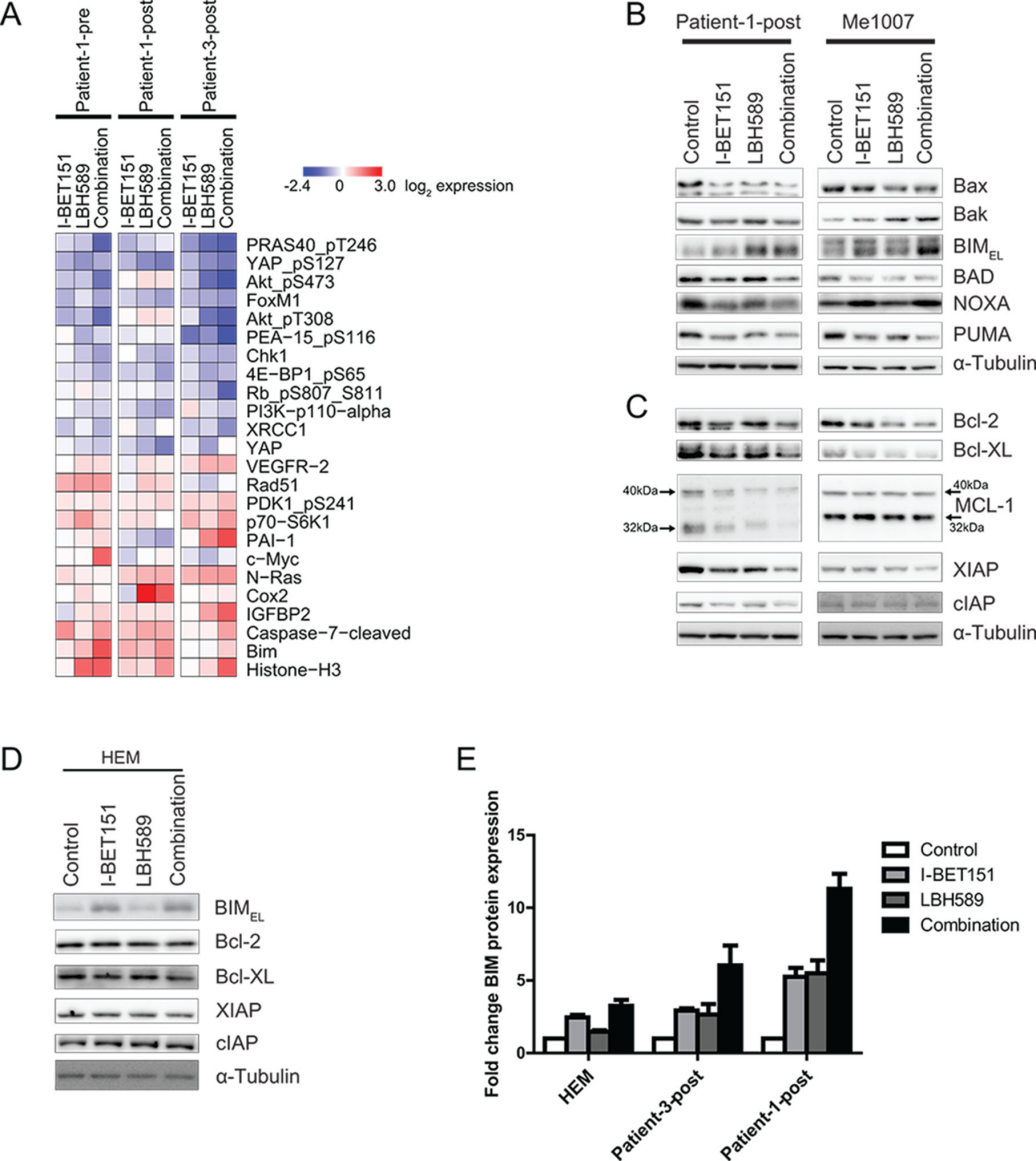
Identification of significant protein expression changes in response to combination treatment **A.** RPPA data show genes that were most changed by combination of I-BET151 and LBH589 after 24 h. Downregulated proteins are represented in blue, whereas upregulated proteins are marked in red. Total protein lysates of Patient-1-post and Me1007 cells, harvested after 24 h upon treatment, were analyzed for changes in protein expression of **B.** pro-apoptotic and **C.** anti-apoptotic proteins by western blot. The large and small isoforms of MCL-1 are indicated by size markers. Expression of α-Tubulin served as internal control. **D.** Changes in protein expression of HEM were analyzed by western blot after 48 h and α-Tubulin was used as loading control. **E.** Fold change of BIM protein expression (± SEM) was quantified from three independent western blots by using ImageJ.

In view of previous studies showing that I-BET151-induced apoptosis was associated with upregulation of BIM [17] we examined the effects of the combination on BIM expression. As shown in Figure 3A BIM mRNA was strongly upregulated by the combination of drugs in the Patient-1-post cell line compared to that induced by the single drugs. BIM was not increased above the levels induced by I-BET151 alone in the Me1007 cell line and may indicate that single drug treatment of I-BET151 induced maximal levels in this cell line. Knockdown of BIM by siRNA in Patient-1-post and Me1007 cells is shown in Figure 3B. The corresponding apoptosis assays indicated that BIM was strongly involved in the induction of apoptosis in both cell lines (Figure 3C), with similar results obtained in a third cell line using a pool of BIM siRNA molecules to reduce BIM expression (Supplementary Figure 3A; 3B). BIM is known to be one of the target genes regulated by the transcription factor FOXO3a. In view of this we silenced FOXO3a expression by siRNA as shown in Figure 3D and found that this resulted in marked inhibition of BIM mRNA expression. Knockdown of FOXO3a also inhibited apoptosis in combination-treated Me1007 cells as shown by the results in Figure 3E. However, apoptosis inhibition was not complete and may indicate other factors were involved in induction of apoptosis. Changes in BIM mRNA and protein levels were not perfectly concordant (see Figure 3A and 4B), suggesting the post-transcriptional mechanisms or changes in BIM stability may also be involved in induction of BIM protein expression.

### Treatment of melanoma cells with the combination of drugs results in downregulation of proteins in the AKT and Hippo/YAP signaling pathways

To elucidate which signaling pathways drive induction of apoptosis by the drug combination, a reverse phase protein array (RPPA) was performed. Cell lines including Patient-1-pre and two of the vemurafenib-resistant cell lines (Patient-1-post, Patient-3 post) were either treated with DMSO, single drug or combination and total protein lysates were prepared after 24 h. A RPPA heatmap of the top 23 proteins changed by combined I-BET151 and LBH589 treatment of all tested cell lines is shown in Figure 4A. This revealed that most of the proteins reduced by combination treatment were in the PI3K/AKT/mTOR pathway (PRAS40_pT246, Akt_pS473, Akt_pT308, 4E_BP1_pS65) or the Hippo/YAP pathway (YAP_pS127, FoxM1, YAP) suggesting an involvement of both pathways in combination-induced apoptosis. Proteins upregulated included the pro-apoptotic BH-3 only protein BIM and cleaved caspase 7.

To validate and extend the RPPA results we performed western blotting on drug treated Patient-1-post and Me1007 cells to measure expression of pro- and anti-apoptotic proteins. BIM was consistently upregulated in cells treated with single drug or combination (Figure 4B). Additionally, there were slight or variable changes in BAK and NOXA expression and varying degrees of downregulation of BAX, BAD and PUMA. There were consistent decreases in expression of Bcl-2, Bcl-XL and of the inhibitor of apoptosis (IAP) protein XIAP and variable changes in MCL-1 and cIAP (HIAP) protein expression (Figure 4C). Changes in BIM, XIAP, Bcl-2 and Bcl-XL were also observed in other cell lines tested (Supplementary Figure 3C). To determine whether these observed changes of pro- and anti-apoptotic protein expression were of relevance in mediating apoptosis, we compared their expression in melanocytes that did not undergo apoptosis when treated with the combination (Figure 1B, Supplementary Figure 2B). Protein expression of pro-apoptotic BIM was increased 3-fold in melanocytes by treatment with the combination (Figure 4D, 4E). However, this was much less than that observed in Patient-1-post and Patient-3-post cells where BIM increased by 6-fold and 11-fold, respectively (Figure 4E). In contrast, protein expression of Bcl-2, Bcl-XL, XIAP and cIAP remained unchanged in melanocytes, suggesting that expression of the anti-apoptotic proteins prevented melanocytes from undergoing apoptosis. Taken together these results indicate that effectiveness of combination treatment on melanoma cells is due to an induction of BIM and a simultaneous decrease in expression of anti-apoptotic proteins Bcl-2, Bcl-XL and XIAP. To give these findings wider relevance we examined The Cancer Genome Atlas (TCGA) data (http://cancergenome.nih.gov/) for the prognostic significance of BIM and cleaved caspase 7 expression. As shown in Supplementary Figure 3D, Kaplan-Meier survival curves associated with TCGA protein data (RPPA) report that overexpression of BIM and cleaved caspase 7 protein is strongly correlated with improved patient survival. We suggest that if the combination of drugs in this study increases BIM and cleaved caspase 7 levels in melanoma patients, as we have observed in mice and *in vitro*, this may be associated with improved clinical outcomes.

### Induction of apoptosis is associated with suppression of AKT and Hippo/YAP signaling pathway

**Figure 5:**
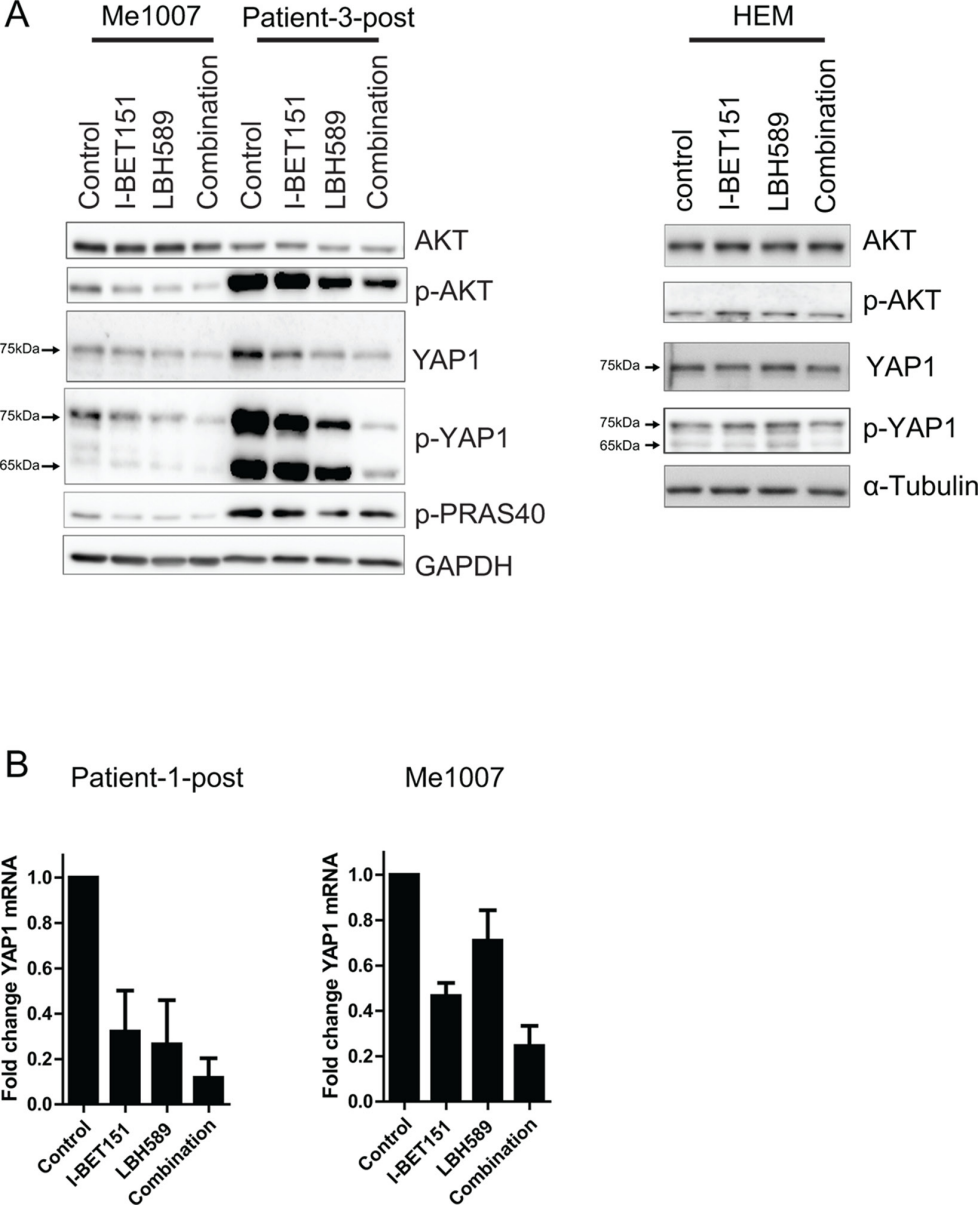
Co-treatment of I-BET151 and LBH589 downregulates AKT and Hippo/YAP signaling pathway **A.** Total protein lysates of drug treated cells (24 h) were analyzed for the expression levels of AKT, its downstream target p-PRAS40 and Hippo/YAP signaling pathway in Me1007, Patient-3-post cells (left panel) and in melanocytes (right panel) by western blot. The phospho-YAP antibody detected an additional smaller splice variant that was not detected by the total-YAP antibody. GAPDH and α-Tubulin served as internal control, respectively. **B.** Downregulation of YAP1 mRNA level was determined by qRT-PCR in Patient-1-post and Me1007 cells after 24 h of drug treatment. Mean (± SEM) of *n* = 2–3 experiments is shown.

To obtain evidence for the importance of downregulation of these pathways in the induction of apoptosis we carried out western blots of key proteins in both pathways. Consistent with the RPPA data, the combination induced strong downregulation of p-AKT, YAP1, p-YAP1 and p-PRAS40 protein expression in Me1007 and Patient-3-post cells and other melanoma cell lines, whereas no change in protein expression could be observed in melanocytes (Figure 5A, Supplementary Figure 4). To analyze the role of YAP1, we further examined YAP1 mRNA expression by quantitative RT-PCR analysis. This revealed a consistent downregulation of YAP1 mRNA expression both in melanoma cell lines Patient-1-post and Me1007 cells, indicating that the combination of I-BET151 and LBH589 reduces YAP1 expression at the transcriptional level (Figure 5B). Taken together these data indicate that the drug combination inhibits two pathways known to be involved in progression of melanoma.

### Combination of I-BET151 and LBH589 inhibits melanoma growth and prolongs survival in a melanoma xenograft model

**Figure 6:**
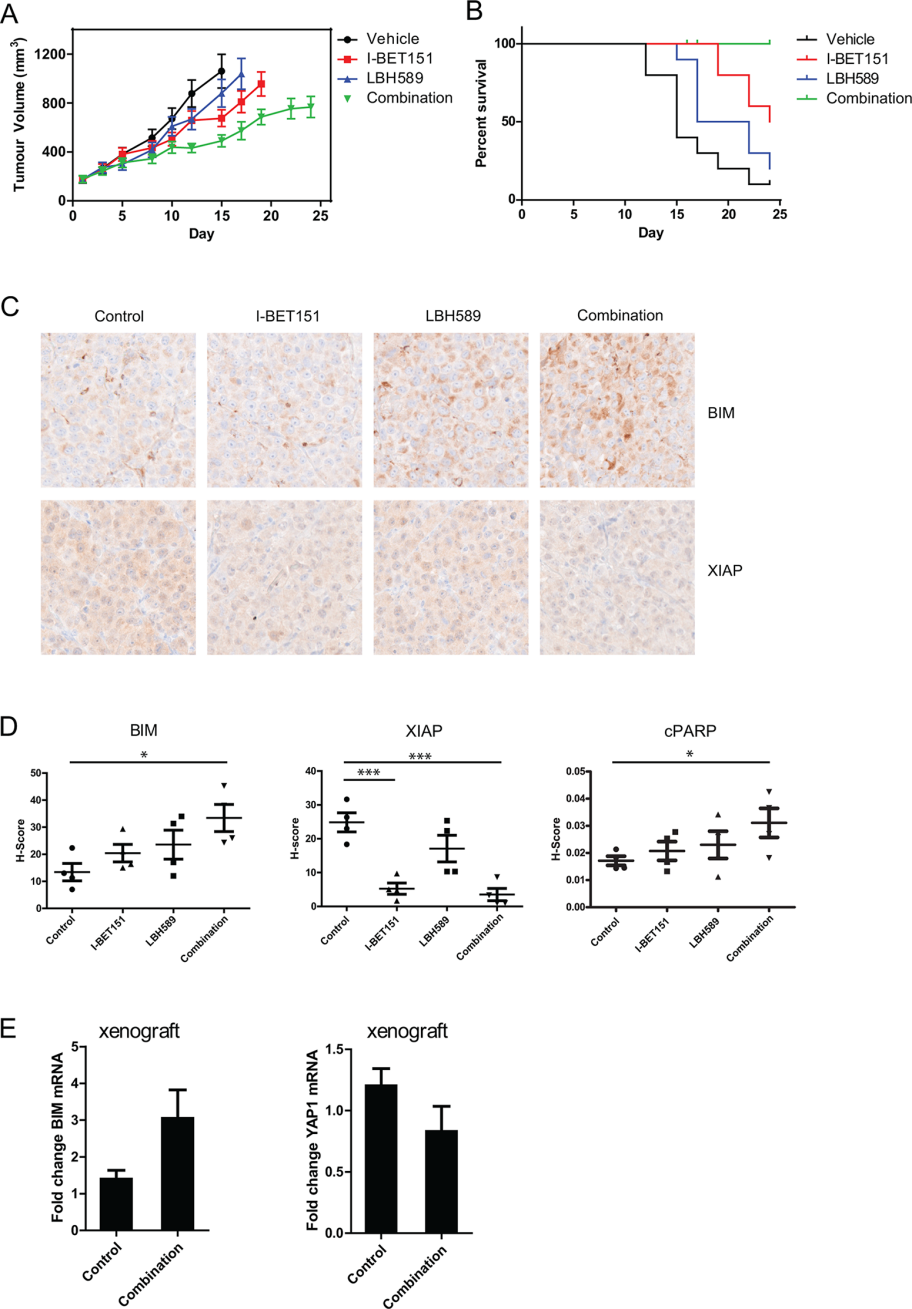
I-BET151 and LBH589 significantly inhibits melanoma growth and prolongs survival in a xenograft mouse model **A.** Tumor volumes and **B.** Kaplan-Meier survival analysis following treatment of mice bearing Patient-1-post tumors with I-BET151 or LBH589 (panobinostat) alone or in combination is shown (*n* = 10 per treatment arm). Average tumor volume is shown until two mice in that treatment arm reached 1200 mm^3^. Compared with treatment of either agent alone, combination of I-BET151/LBH589 treatment significantly reduced tumor growth (vehicle vs. combination 65.4%, *p* < 0.001) and **B.** prolonged survival of mice (where survival of mice is defined as time to reach a tumor volume of 1200 mm^3^). **C.** Immunohistochemical analysis of BIM and XIAP levels in xenografts from the survival study are shown. **D.** Expression levels of BIM, XIAP and cleaved PARP were quantified in four separate xenografts per treatment (mean ± SEM). Compared to control mice, combination treatment lead to high tumor expression of BIM ( *p* = 0.015) and cPARP (0.047) and reduced XIAP (*p* = 0.0007) expression. **E.** A separate short term experiment measured BIM and YAP mRNA expression level of xenograft tumor tissue following 3 h of treatment. Mean expression levels (± SEM) of *n* = 3–4 mice per group is presented.

To determine whether combination of I-BET151 and LBH589 is effective *in vivo*, a subcutaneous xenograft mouse model engrafted with Patient-1-post cells was used, and mice treated with either vehicle control, drugs alone or in combination (ten mice per treatment arm). As shown in Figure 6A, treatment with I-BET151 or LBH589 alone inhibited tumor growth by 44.3% (*p* < 0.01, ANOVA, Dunnett's post-hoc test) and 22.3% (ns), respectively on day 15 when compared to vehicle treated tumors. Combined treatment with I-BET151 and LBH589 reduced tumor growth further by 65.4% (*p* < 0.001). Kaplan-Meier analysis of survival of the mice revealed that, as compared to vehicle control, treatment with I-BET151 (*p* < 0.01, Mantel Cox log rank test) but not LBH589, prolonged the survival (as defined by time to an ethical tumor volume endpoint) of mice. Combined treatment with I-BET151 and LBH589 further prolonged survival as compared with I-BET151 alone (Figure 6B) (*p*-value < 0.05). These results were extended using clonogenic assays on Me1007, Mel-RM and Patient-1-post cells which showed that the combination was superior to that of individual drugs (Supplementary Figure 5A; 5B). Consistent with *in vitro* analysis, immunohistochemical staining confirmed induction of BIM and cleaved PARP and a decrease of XIAP in tissue sections of xenografts harvested from mice in the survival study following euthanasia. Again, the strongest effects on BIM, cPARP and XIAP expression level were observed in the combination-treated cells (Figure 6C, 6D). Moreover a separate experiment investigating short term effects on xenografts following drug treatment showed induction of BIM mRNA and a reduction of YAP1 mRNA expression in combination-treated tumor tissue xenografts (Figure 6E). Taken together, these findings indicate that combined treatment with I-BET151 and LBH589 inhibits tumor growth *in vivo* and prolongs survival of mice with melanoma xenografts.

## DISCUSSION

HDACs are frequently components of protein complexes that repress gene expression by removing acetyl groups from histones [5]. BET proteins on the other hand are components of complexes that target transcription factors to acetylated histones particularly at sites of so called super enhancers [20, 21]. We reasoned that HDAC inhibitors (HDACi), by increasing acetylation of histones, would increase interaction with BET proteins and increase the sensitivity of cells to BET protein inhibitors. Our results support this hypothesis and the combination of these drugs was more effective than either drug alone in inducing apoptosis and arresting tumor growth in melanoma. Apoptosis was mediated by classical mitochondrial and caspase dependent pathways and involved upregulation of the BH3 pro-apoptotic BIM. This was accompanied by downregulation of a range of anti-apoptotic proteins such as Bcl-2, Bcl-XL and XIAP.

These results are consistent with our previous studies on the individual inhibitors which showed that HDAC inhibitors resulted in upregulation of several pro-apoptotic proteins and downregulation of Bcl-XL and XIAP [5, 11, 12]. Similarly we found that I-BET151 induced BIM expression (but not NOXA or PUMA) as well as downregulation of Bcl-2, Bcl-XL and XIAP [17]. In addition to effects on apoptosis, cells cultured in the drug combination underwent morphological changes of flattening and spreading as well as cell cycle arrest. Single drug treatment with I-BET151 induced G_0–1_ arrest [17, 22, 23]. In contrast, LBH589 treatment has been described to mediate G_0–1_ and/or G_2-M_ arrest in different tumors [24–27] by induction of the cell cycle inhibitor p21 [27]. In the present studies the combination of LBH589 and I-BET151 caused enhanced expression of p21 compared to each drug alone and an increase in the number of cells with 4N DNA content, suggestive of arrest predominantly in the G_2-M_ phase.

Similarities in the proteins targeted by the two drugs are perhaps not unexpected as both drugs act by targeting acetylated histones. Previous studies have also reported that inhibitors of BET proteins and histone deacetylases share transcriptional signatures for genes involved in cell cycle and apoptosis [28]. Studies on a wider range of proteins by RPPA revealed strong downregulation of proteins in the AKT pathway that was not evident in studies on melanocytes. This was reported by others [29, 30] and results from acetylation of Hsp90 and thereby loss of its chaperone activity against proteins like AKT, EGFR and STAT3 [24, 30, 31]. Both I-BET151 treatment alone and in combination with LBH589 increased BIM mRNA, suggesting a transcriptional mechanism driving the increase in BIM protein levels.

Given that AKT is a major regulator of cell survival and apoptosis [32], its downregulation could promote apoptosis by a number of mechanisms. This includes phosphorylation of the transcription factor FOXO3a which prevents its entry into the nucleus and thereby its upregulation of BIM [32]. Our data showing upregulation of BIM is consistent with this and further supported by studies showing that knockdown of FOXO3a prevented upregulation of mRNA for BIM by the drug combination. The exact involvement of FOXO3a requires further study however as while both siRNA molecules reduced BIM mRNA expression by a similar amount, one was less effective at preventing apoptosis. A number of factors could cause this, including differential effectiveness of the siRNA to reduce FOXO3a protein levels and BIM independent effects of FOXO3a. Induction of BIM is certainly not the only mechanism by which the drug combination induces cell death. This is demonstrated by the fact that knockdown of BIM by siRNA failed to completely prevent apoptosis, indicating that other factors were involved such as the downregulation of the anti-apoptotic proteins Bcl-2, Bcl-XL and XIAP. This interpretation was consistent with studies on melanocytes that had no decrease in anti-apoptotic proteins and did not undergo cell death despite a 3-fold increase in BIM protein expression. Sustained PI3K activity was also reported to protect melanocytes from apoptosis [33, 34]. The importance of changes in anti-apoptotic proteins in the induction of apoptosis by HDACi was supported by previous studies showing that overexpression of Bcl-2 and Bcl-XL suppressed apoptosis induced by the histone deacetylase inhibitors LAQ824 and LBH589 [35]. Several questions raised from the RPPA studies remain unanswered. One is the role of strong downregulation seen of YAP and phosphorylated YAP induced by the combination in induction of apoptosis. These proteins are in the Hippo pathway which appear important in progression of uveal melanoma. Dephosphorylation of YAP by the mutant Gq11 component of G protein coupled receptors allows its entry into the nucleus [36]. On entry into the nucleus it acts as a co-factor with transcription factors such as TEADs and SMADs to induce genes involved in cell cycle regulation such as FOXM1 and Cyclin D1 [37] and to inhibit apoptosis by regulation of IAP proteins [38]. It was notable in the present studies that these proteins were downregulated in melanoma cells treated by the combination of HDAC and BET protein inhibitors and questions whether these changes contributed to apoptosis. Downregulation of the YAP proteins appeared to be transcriptional as shown by the marked downregulation of YAP mRNA and not due to binding to intra-cytoplasmic proteins that regulate entry into the nucleus, such as 14-3-3 proteins [38]. Given YAP's involvement in uveal melanoma further studies appear warranted to assess effects of the combination on uveal melanoma.

Another question is why many changes seen in melanoma cells are not seen in normal melanocytes. Similar findings were reported in studies on isogenic normal and transformed cells treated with HDACi which showed a tumor selective pro-apoptotic gene signature [39]. Presumably, epigenetic mechanisms that are targeted by the two inhibitors have been selected to protect the melanoma cells against apoptosis and in maintaining their proliferation. These questions are the subject of ongoing studies.

The availability of protein arrays in the The Cancer Genome Atlas (TCGA) data allowed us to examine whether the changes in proteins seen in our studies with this combination of drugs might have prognostic significance in larger populations of patients. This showed that high levels of BIM and cleaved caspase 7 were highly associated with improved survival, implying that changes induced by the combined drugs may have beneficial effects on survival. These conclusions were supported by the results of the studies on melanoma xenografts in NOD/SCID mice which showed that the combination of I-BET151 and LBH589 significantly improved survival of the mice challenged with the vemurafenib-resistant Patient-1-post melanoma cell line compared to either drug alone. The same synergistic effects of the drug combination were seen in colony formation assays. *In vivo* responses were accompanied by similar changes in protein levels of BIM and XIAP as found *in vitro*. The present finding of synergistic effects between these two classes of drugs are consistent with similar findings in studies on human acute myelogenous leukemia cells showing that the combination of the BET bromodomain inhibitor JQ1 and HDACi LBH589 was superior to treatment with either agent alone in inducing apoptosis and survival of NOD/SCID mice engrafted with acute myelogenous leukemia cells [23].

In summary this study shows that combined inhibition of HDAC and BET proteins has synergistic effects in the treatment of melanoma *in vitro* and *in vivo* which are associated with more marked increases in upregulation of the pro-apoptotic protein BIM and downregulation of the anti-apoptotic proteins Bcl-2, Bcl-XL and XIAP than seen with the single drugs alone. The treatment combination was associated with marked downregulation of the AKT pathway and of YAP proteins in the Hippo pathway. Further studies are needed to understand how these pathways are regulated epigenetically. These preclinical studies provide a basis for considering combinations of these epigenetic inhibitors in new treatments for melanoma particularly those resistant to BRAFi.

## MATERIALS AND METHODS

### Cell lines

Melanoma cell lines Patient-1-pre, Patient-1-post, KMJR138, Patient-3-post, Me1007 and Mel-RM have been described previously [40]. Patient-1-pre, Patient-1-post, and Patient-3-post are patient cell lines established before treatment or during relapse from treatment with vemurafenib, labeled “pre” and “post”, respectively, as described elsewhere and are all BRAF^V600E^ mutant cell lines [41]. Cells were cultured in Dulbecco's modified Eagle medium (DMEM) containing 10% fetal calf serum (FCS) and 1% Penicillin/Streptavidin (AusGeneX, Brisbane, Australia). Human melanocytes (HEM) were purchased from Life Technologies (Carlsbad, CA, USA) and cultured in Media 254 complemented with Human Melanocyte Growth Supplement (Gibco, Victoria, Australia).

### Chemicals and transfection

I-BET151 was supplied by GlaxoSmithKline (Brentford, UK). LBH589 (Panobinostat) was purchased from Selleckchem (Houston, TX, USA). Cells were treated with either with 2 μM I-BET151, 30 nM LBH589 or combination and control cells were treated with DMSO. For inhibition of caspase activity, 10 μM of Q-VD-OPh (SM Biochemicals, Anaheim, CA, USA) was added to culture medium 30 minutes before other additional treatment. For gene knockdown studies, cells were transiently transfected with siRNA of BCL2L11 (siRNA#1: SI04951968; siRNA #2: SI02655359 Qiagen, Venlo, Netherlands), FOXO3a (siRNA #1: SI04916366, siRNA #2: SI04916387) or a non-silencing control (1027281, Qiagen) using Lipofectamine RNAiMax (Invitrogen, Carlsbad, CA, USA) according to the manufacturer's protocol. Cells were transfected 24 h before being drug-treated for a further 48 h.

### Analysis of cell death, synergy, cell cycle and mitochondrial membrane potential

Percentage of apoptotic cells was determined using Annexin-V/Propidium Iodide (PI) staining following the manufacturer's instructions (Becton Dickinson, Franklin Lakes, NJ, CA, USA) and analyzed employing a Becton Dickinson FACSCalibur flow cytometer and CellQuest Pro software. Synergy of drug interactions was calculated using the fixed ratio/combination index method [42]. Apoptotic cells were measured by Annexin-V/PI staining and combination index (CI) calculated using CalcuSyn software Version 2.1 (Biosoft, Cambridge, UK). CI with values < 1.0 indicate a synergistic interaction of both drugs in the combination and CI values < 0.5 indicate strong synergy. For cell cycle analysis, cells were stained with PI and analyzed by flow cytometry. Cell cycle was fitted to viable cells using ModFit LT software (Verity Software House). Changes in mitochondrial membrane potential (ΔMOMP) were stained with the membrane determined by staining cells with JC-1 as described by the manufacturer (Molecular Probes, Eugene, OR, USA) followed by flow cytometry analysis.

### Reverse phase protein array (RPPA)

Cell lines were either treated with DMSO, 2 μM I-BET151, 30 nM LBH589 or combination for 24 h. Lysates were prepared as recommended by MD Anderson Cancer Center (Houston, Texas, USA), arrayed on nitrocellulose-coated slides, probed for a standard list of antibodies at the MD Anderson Cancer Center and results were quantified and normalized using their procedure [43]. Genes with insufficient signal were filtered out. For heatmaps, differential protein expression was calculated by subtracting log2 transformed protein levels of control (DMSO) treated cells from treated cells.

### Western blotting

Tumor cells were lysed in RIPA buffer. Proteins were separated by SDS-PAGE, electroblotted onto nitrocellulose membranes and probed with the following primary anti-human antibodies: BIM (C34C5), Bax (2772), PUMA (4976), caspase 7 (9492), caspase 9 (9502), AKT (9272) and phospho AKT (pS473, 9271), pPRAS40 (pThr 246, 2997) all from Cell Signaling Technology (Cambridge, UK), Bcl-2 (C-2, sc-7382), Bcl-XL (H-5, sc-8392), Bak (G-23, sc-832), caspase 3 (sc-7148), PARP (F-2, sc-8007), GAPDH (sc-32233), YAP1 (sc-15407) from Santa Cruz (Santa Cruz, CA, USA), anti-MCL-1 (559027) and anti-XIAP, (610716), BAD (610392) from BD Bioscience (San Jose, CA, USA), NOXA (114C307.1) from Imgenex (Littleton, CO, USA), pYAP1 (pS127, ab76252) from abcam (Cambridge, UK), cIAP/HIAP-2 (AF 8181) from R&D (Minneapolis, MN, USA) and alpha-Tubulin (B-5-1-2) from Sigma (St. Louis, MO, USA). After washing, membranes were probed with the appropriate secondary antibodies conjugated to horseradish peroxidase. Antibody binding was visualized using Clarity™ Western ECL substrate (Bio-Rad, Hercules, CA, USA). Band intensities were quantified by using ImageJ software.

### qRT-PCR

RNA was extracted from cell lines using RNeasy Plus mini prep kit (Qiagen), quantitated using a Nanodrop (Thermo Scientific, Wilmington, DE, USA) and 1 μg RNA reverse transcribed with SuperScriptIII (Invitrogen). cDNA was amplified on AB7900 (Applied Biosystems, Mulgrave, VIC) using Universal PCR Master Mix and Taqman probes specific for BIM (Hs00708019_s1), YAP (Hs00371735_m1), Foxo3a (Hs00818121_m1) and normalized to levels of endogenous 18S (Hs99999901_s1) (Applied Biosystems).

### *In vivo* experiments

All animal experiments were performed with approval from the Peter MacCallum Cancer Centre Animal Experimentation Ethics Committee. Female NOD/SCID mice (Animal Resources Centre, Western Australia) were injected subcutaneously into the flank with 4 × 10^6^ Patient-1-post cells in 50% Matrigel. Once tumors had grown to approximately 130 mm^3^, mice were randomized into four groups of ten mice and each group administered 15 mg/kg I-BET151 via oral gavage daily, 4 mg/kg LBH589 via ip injection days 1–5 each week, drug vehicles or the combination of both drugs. Tumor volumes were determined thrice weekly and mice were euthanized once tumors exceeded 1200 mm^3^ or the animals showed signs of distress. Following euthanization, tumours from 4 mice per treatment arm were randomly chosen for IHC analysis. Additionally, a separate, short term experiment was performed in which mice harbouring xenograft tumours were given a single dose of each drug or combination before being euthanized and sacrificed after 3 h. Fresh frozen tumour sections were collected for mRNA analysis. This experiment was performed on 3–4 mice per treatment group.

### Immunohistochemistry

Immunohistochemistry was performed on formalin fixed, paraffin embedded xenograft tissue and then constructed into a tissue microarray (TMA). Where feasible three tissue cores 1 mm in diameter were taken from the donor paraffin block using the marked section as a reference and arranged in a blank paraffin block utilising a TMArrayer™ (Pathology devices, Westminster, USA). Sections were incubated with the respective primary antibodies at the following dilutions: BIM (1:100, CS2933), cPARP (1:100, CS9541), XIAP (1:200, BD610716). Antibody detection was performed on a Dako Autostainer Plus (Dako, Glostrup, Denmark) using the MACH 3 visualization kit (M3R530 and M3R531, Biocare Medical, Concord, CA, USA) according to the manufacturers’ protocols.

### Quantitative image analysis

TMA sections were scanned using the multispectral Vectra slide scanner (PerkinElmer, Waltham, MA, USA). Brightfield images were captured at 20 nm intervals from 420 nm to 720 nm at 4x and 20x magnifications for low power and high power images respectively. The captured images were analyzed using the quantitative InForm image analysis software [44]. Dependent on the immunostain present, the software could then measure and score the intensity of the immunostaining on a cell by cell basis and an H-score (scale 0–300) which considers both the intensity and percentage of cells staining at each intensity bin (0 + to 3 +) [45] or percentage positive/negative ratio was reported.

### Statistical analysis

Graphs are presented as mean ± SEM and are representative of three experiments unless otherwise stated. Statistical significance was determined by unpaired, two-tailed student's *t*-test. *indicates *p* < 0.05, **indicates *p* < 0.01 and ***indicates *p* < 0.001.

## SUPPLEMENTARY DATA


