# Supplementary material for: Combining BET and HDAC inhibitors synergistically induces apoptosis of melanoma and suppresses AKT and YAP signaling
Source: Oncotarget. 2015 Jun 5;6(25):21507–21. doi: 10.18632/oncotarget.4242 (PMC4673282; doi:10.18632/oncotarget.4242)
Supplement: Supplementary file 1 [file oncotarget-06-21507-s001.pdf]

SUPPLEMENTARY DATA

siRNA transfection

To knockdown BIM, Patient-3-post cells were transiently transfected with ON-TARGET plus SMARTpool for human BCL2L11 (siRNA#3:L-004383-00-0005) and a non-silencing control (D-001810-10-05) using Dharmafect1 according to the manufacturer's protocol (all purchased from Dharmacon, Waltham, MA, USA). Cells were transfected 24 h before being drug-treated with 2 µM I-BET151, 30 nM LBH589, combination or diluents only. After 48 h cells were harvested for western blot analysis and Annexin-V/PI staining.

Colony forming assay

A clonogenic assay was performed to determine cell survival after treatment with I-BET151 and LBH589. Melanoma cells were seeded in 6 well plates (from 50–400 cells per well) in the presence of 2 µM I-BET151, 30 nM LBH589 combination or diluent only. After 48 h, media was replaced after careful washing with PBS and plates were incubated for 8-10 days at 37°C. Cells were fixed and stained with 0.5% crystal violet in 20/80 methanol/water for 30 min. The numbers of colonies with > 30 cells were counted and the survival fraction was calculated as described [1]. Mean (± SEM) of $n = 5$ experiments is shown.

REFERENCES

1. Franken NA, Rodermond HM, Stap J, Haveman J, van Bree C. Clonogenic assay of cells *in vitro*. Nat Protoc. 2006; 1:2315–2319.

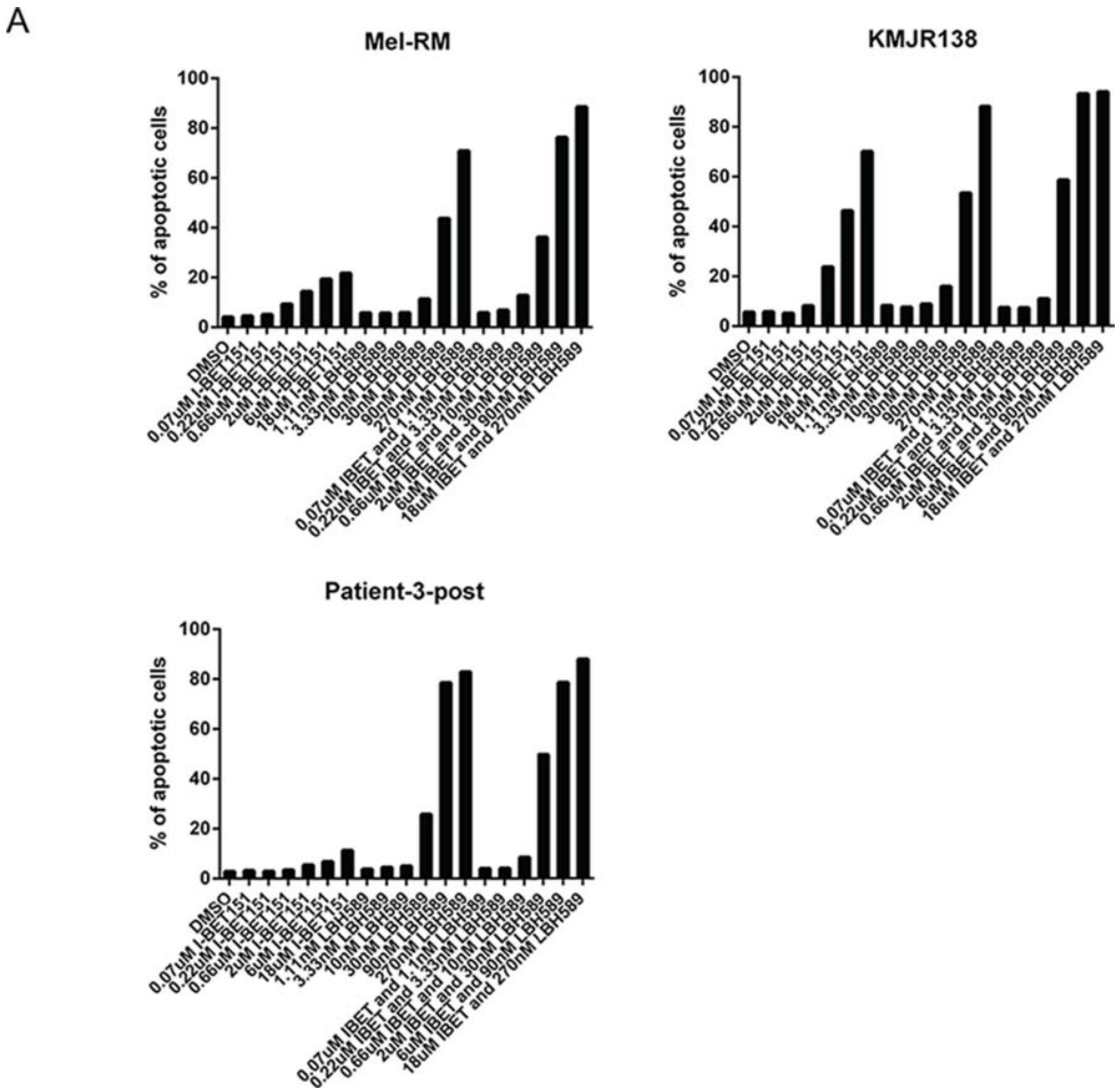

Supplementary Figure S1: Pan-HDAC inhibitor LBH589 and BET protein inhibitor I-BET151 cause dose-dependent induction of apoptosis. A. The indicated cell lines were treated with increasing concentrations of LBH589 and I-BET151 alone or in combination, for 48 h and apoptosis measured by Annexin-V/PI staining followed by flow cytometry analysis. Representative results from one experiment are shown.

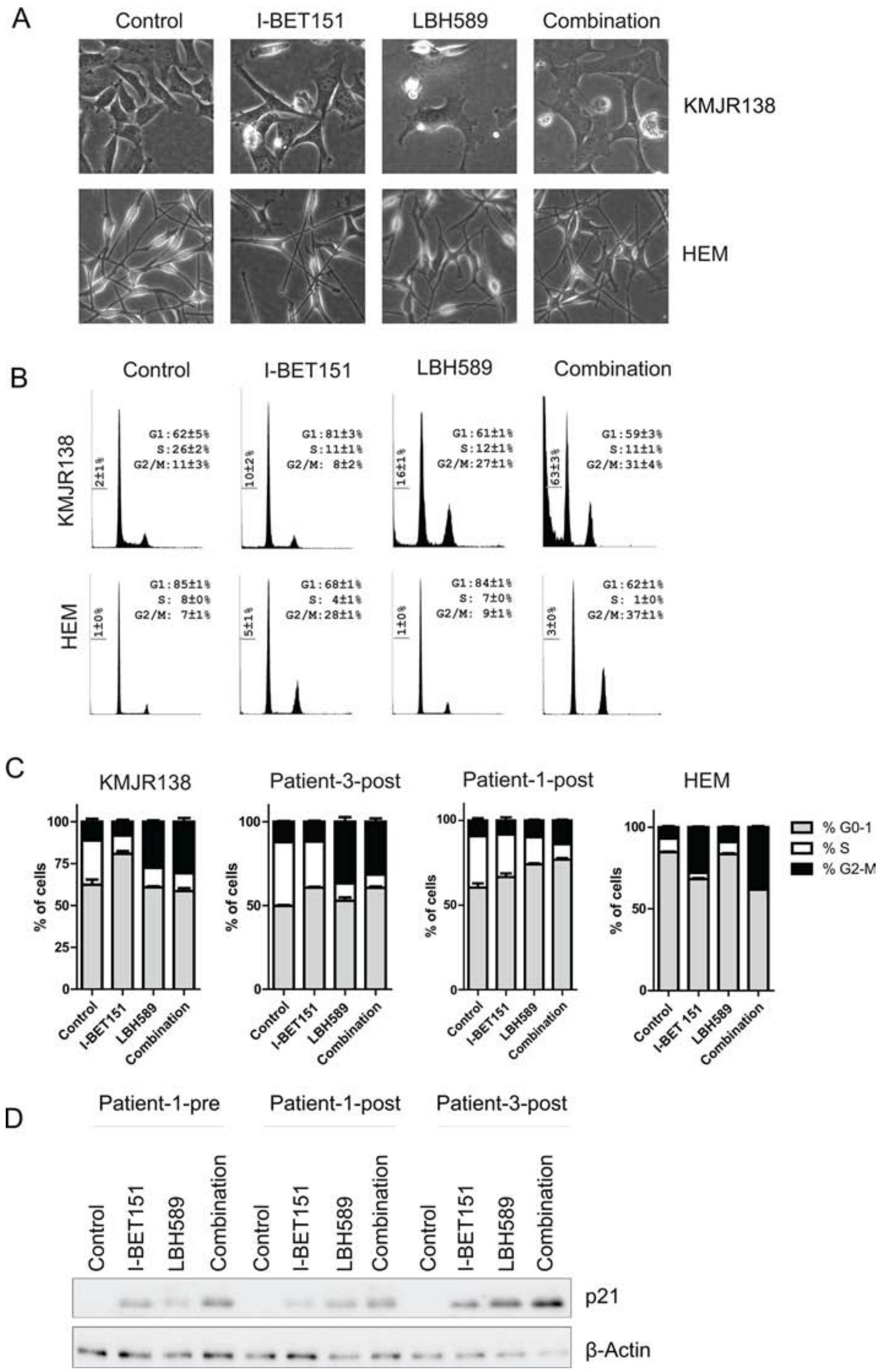

Supplementary Figure S2: Combined treatment with I-BET151 and LBH589 induces apoptosis and cell cycle growth arrest in melanoma cells. A. Microscopic images of KMJR138 cells and melanocytes treated with 2 μM I-BET, 30 nM LBH589, combination or vehicle for 48 h are presented. Cell cycle analysis was performed after treatment as described above by flow cytometry and after staining cells with PI. Cell cycle distribution of viable cells was calculated using Modfit LT software and is shown as a representative cell cycle histogram **B.** and a bar histogram **C.** Mean (± SEM) of one experiment performed in triplicates is shown. Percentage of cells in sub-G1 (apoptotic cells) was determined by PI staining and flow cytometry analysis. **D.** A representative western blot for indicated melanoma cell lines detecting protein expression p21 is shown. β-actin was used as internal control.

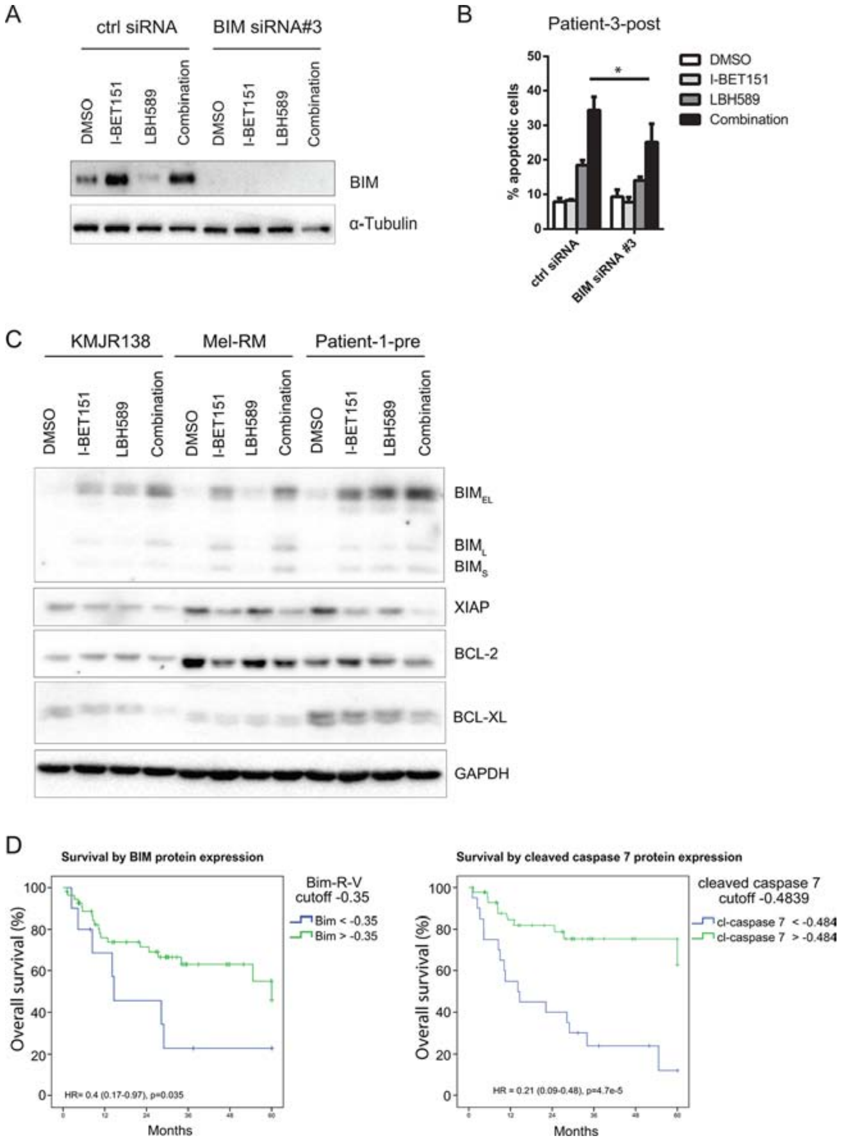

Supplementary Figure S3: Increased BIM expression causes cell death in response to combination treatment with I-BET151 and LBH589 and high expression of BIM and cleaved caspase 7 protein is correlated with improved patients overall survival. A. BIM expression was ablated in Patient-3-post cells using a Dharmacon smart-pool for 24 h and cells treated 2 µM I-BET151 and 30 nM LBH589 for a further 48 h. Western blotting showed siRNA treatment efficiently reduced BIM protein levels and **B.** reduced the induction of cell death as measured by Annexin-V/PI staining and flow cytometry. **C.** Changes in Bcl-2 family members were measured in the indicated cell lines after treatment with 2 µM I-BET151 and 30 nM LBH589 for 24 h. **D.** Kaplan-Meier curves from reverse phase protein array (RPPA) protein data sourced from the TCGA data repository show the effect of BIM (left panel) and cleaved caspase 7 (right panel) overexpression on patient survival.

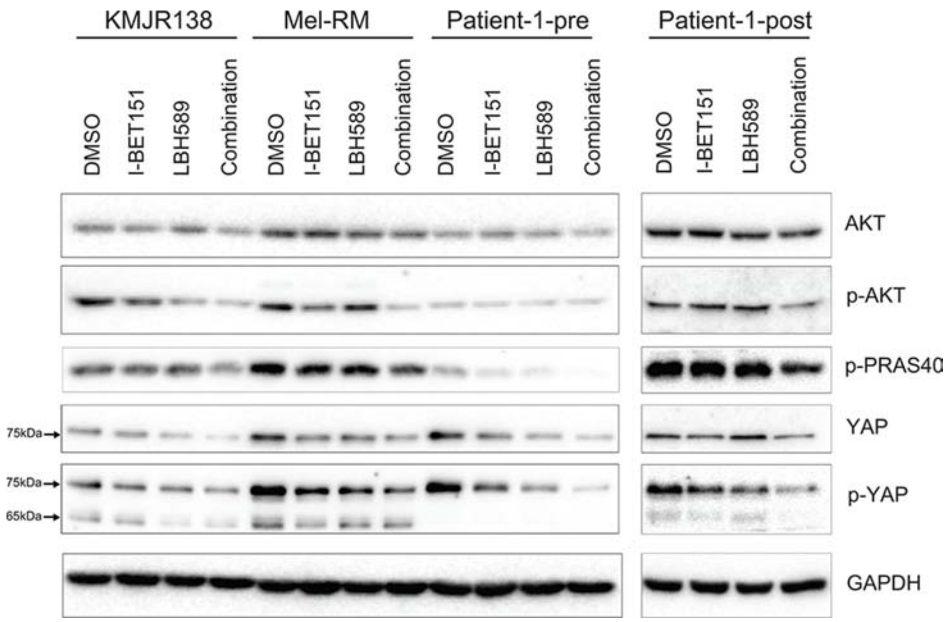

Supplementary Figure S4: Combination treatment with I-BET151 and LBH589 reduces phosphorylation of AKT and YAP. The indicated cell lines were treated with 2 µM I-BET151 and/or 30 nM LBH589 for 24 h and AKT, *p*-AKT, *p*-PRAS40, YAP and *p*-YAP protein expression was measured. Patient-1-post cells have a low levels of *p*-AKT expression and the presented blot is from a longer exposure than the other cell lines. The phospho-YAP antibody detected an additional smaller splice variant (65 kDa) that was not detected by the total-YAP antibody. GAPDH was used as internal control.

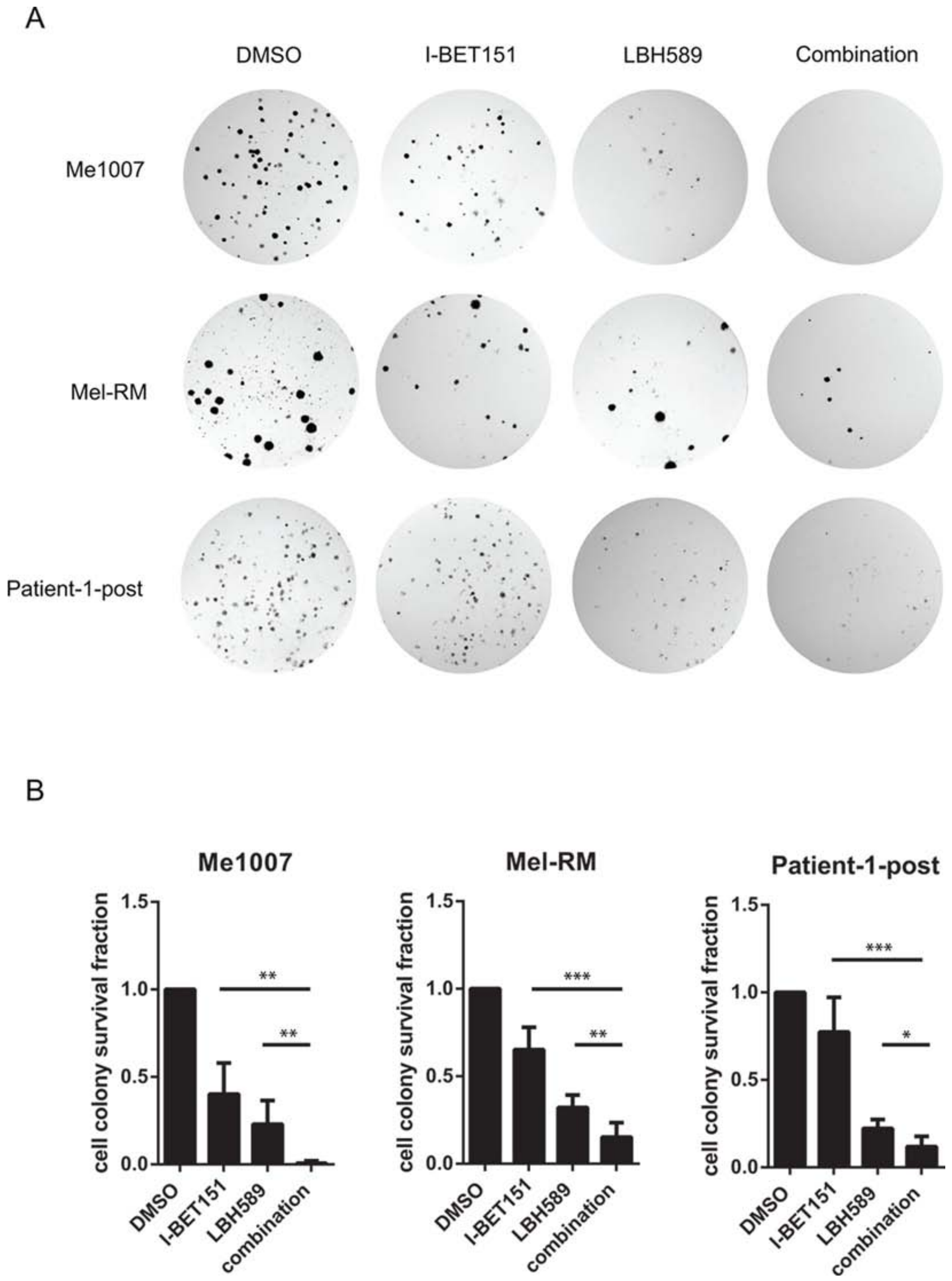

Supplementary Figure S5: Combining I-BET151 and LBH589 more effectively reduces melanoma colony formation than single drug treatment. A. A clonogenic assay was performed on the indicated cell lines and **B.** cell colonies over ~ 30 cells were counted. The results showed that combination drug treatment reduced colony formation more effectively than single drug treatment. Mean expression levels (± SEM) of *n* = 5 experiments are presented.
